# Beyond apoptosis: Implications of annexin-V binding to myeloid cells in DARC (Detection of Apoptosing Retinal Cells) imaging

**DOI:** 10.4103/NRR.NRR-D-25-00182

**Published:** 2025-09-03

**Authors:** Kiyoharu J. Miyagishima, Francisco M. Nadal-Nicolás, Wei Li

**Affiliations:** Retinal Neurophysiology Section, National Eye Institute, National Institutes of Health, Bethesda, MD, USA

*In vivo* imaging of neurodegenerative diseases provides valuable insights into disease mechanisms and potential therapeutic interventions. Many ocular diseases are closely linked to neurodegenerative conditions affecting the brain, making the eye a unique and accessible model for studying these disorders. The transparency of eyes allows researchers to monitor disease progression non-invasively, offering a window into neural health. In particular, the retina serves as a critical platform for studying neurodegeneration and exploring therapeutic strategies that may translate across different diseases.

Glaucoma is the leading cause of blindness worldwide. It is clinically characterized by damage to the optic nerve, a bundle of axons that extend from the back of the eye and transmit visual information to the brain. These axons originate from retinal ganglion cells (RGCs), which integrate and relay visual signals, making them a critical component of the visual pathway. The progressive degeneration of RGCs and their axons occurs insidiously, with symptoms often becoming noticeable only in the later stages of the disease. Untreated, glaucoma leads to irreversible vision loss, underscoring the urgent need for early detection and effective disease management. While there are several different types of glaucoma, they all share a common feature: damage to the optic nerve – often due to buildup of intraocular pressure due to impaired drainage of aqueous humor in the anterior part of the eye. This increased pressure can contribute to optic nerve damage, making intraocular pressure control a primary target for glaucoma treatment.

Despite ongoing efforts to develop neuroprotective treatments, the disease remains incurable. However, medications and surgical interventions can slow or halt its progression, making early diagnosis crucial for preserving vision. Effective disease management relies on accurately assessing a patient’s current status (disease staging) and estimating the risk of progression based on key factors, including ocular measures (intraocular pressure, visual field loss, cup-to-disk ratio, corneal thickness), demographic factors (age, family history, race/ethnicity), vascular health, myopia, and systemic conditions (diabetes, migraines). Unfortunately, existing tools for evaluating both present disease state and future risk remain imperfect, underscoring the need for innovative diagnostic technologies to improve patient outcomes.

In 2004, the Detection of Apoptosing Retinal Cells (DARC) technique was developed to fluorescently label and image dying cells in the retina using annexin-V (Cordeiro et al., 2004). Annexin-V has long been a standard tool for detecting apoptotic cells *in vitro* (Koopman et al., 1994). The principle behind this method is straightforward: in healthy cells, the phospholipid bilayer is organized such that phosphatidylserine remains confined to the inner membrane. However, during apoptosis, phosphatidylserine becomes exposed on the outer membrane, serving as an “eat me” signal for macrophages and other phagocytes. While originally recognized for its role in apoptosis detection, annexin-V has recently been more broadly associated with cellular stress (Draeger et al., 2011; Klement et al., 2012). In principle, by binding to this exposed phosphatidylserine, fluorescently labeled annexin-V enables the visualization of apoptotic and stressed cells *in vivo*, offering a potential tool for early disease detection and monitoring neurodegeneration.

**Figure 1:**
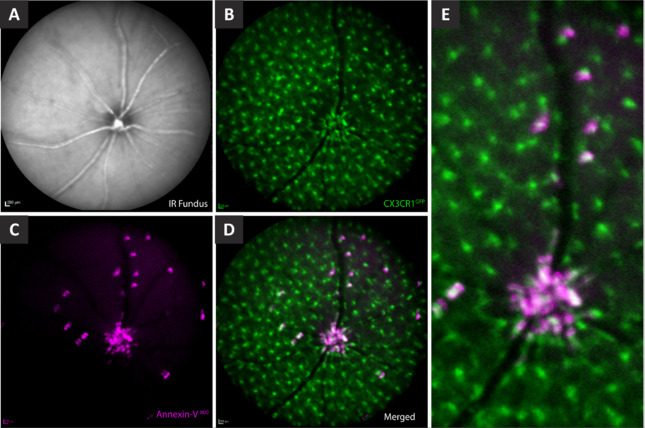
*In vivo* detection of annexin-V^+^ cells in the naïve retina of CX3CR1-green fluorescent protein (GFP) mice. (A) Infrared (IR) Fundus-image showing the retinal vasculature and optic nerve head. (B) GFP-fluorescent microglia (green) in their resting state exhibiting ramified morphology and are evenly distributed across the retina. (C) Annexin-V^+^ labeled cells (magenta) predominantly concentrated at the optic nerve head. (D) Merged image of B and C demonstrating that a subpopulation of GFP-fluorescent microglia co-localize with annexin-V, indicating that not all annexin-V^+^ cells are apoptotic. (E) Magnified optic disc from D. Reprinted with permission from Miyagishima et al. (2024).

Our recent work demonstrated that, at least in the mouse retina, there is heterogeneity in the labeling of cells with annexin-V (Miyagishima et al., 2024). Interestingly, we found that the annexin-V positive cells visible on the confocal scanning laser ophthalmoscope were not exclusively apoptotic but also labeled a subpopulation of myeloid cells expressing a CX3CR1-GFP reporter (**Figure 1**). Myeloid cells in the eye are immune cells responsible for maintaining homeostasis. These include microglia, which reside in different layers of the retina, and hyalocytes, which are in the vitreous. To investigate the relationship between myeloid cells and annexin-V labeling, we pharmacologically depleted myeloid cells using PLX5622, a colony-stimulating factor 1 receptor inhibitor essential for their survival. This depletion resulted in the complete loss of annexin-V labeling in the retina. Upon cessation of PLX5622 treatment, myeloid cells repopulated the retina, and annexin-V labeling was restored, confirming that the brightly labeled annexin-V-positive cells belonged to the myeloid lineage.

**Figure 2:**
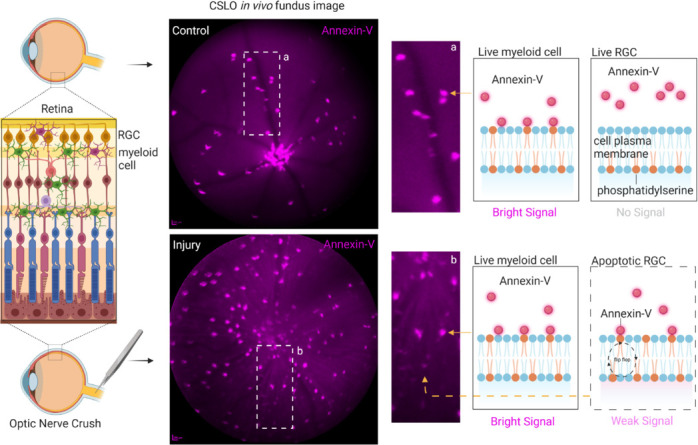
Schematic representation of apoptotic and non-apoptotic cells in the retina labeled with fluorescently conjugated Annexin-V. CSLO images reveal sparse Annexin-V labeling in control retinas, corresponding mainly to myeloid cells. After optic nerve injury, there is a marked increase in bright Annexin-V^+^ cells and numerous weaker puncta, likely reflecting apoptotic cells. A schematic interpretation of Annexin-V labeling in both conditions is shown on the right. Created with BioRender.com. CSLO: Confocal scanning laser ophthalmoscopy; RGC: retinal ganglion cells.

Notably, in the context of optic nerve crush injury, myeloid cell depletion revealed a previously undetectable population of weaker labeled apoptotic RGCs on the confocal scanning laser ophthalmoscope. This suggests that annexin-V labeling of these myeloid cells may obscure apoptotic RGCs under normal conditions. Thus, annexin-V labeling *in vivo* may not be a straightforward indicator of apoptosis highlighting the need for a more nuanced interpretation of DARC imaging results (**Figure 2**).

Further supporting this interpretation, there have been several reports over the years that annexins bind to other immune cells (Dillon et al., 2001) and that some immune cells even express annexins (Fan et al., 2004; Bollinger et al., 2020), which can mediate or modify cellular functions such as phagocytosis. At least in the eye, apoptotic cells labeled with annexin-V appear to be obscured by the brighter signals associated with the labeling of this myeloid cell population. In the optic nerve crush model, the increase in the number of annexin-V^+^ myeloid cells follows the time course of apoptosis, albeit delayed in time by a few days. This cumulative response is likely the driving factor behind the success to date of a naïve interpretation of DARC fluorescence. Assuming this is true in other organs and tissues, the *in vivo* use of annexin-V may provide a unique opportunity to investigate the role of these cells in immune surveillance and maintenance of homeostasis under physiological conditions, as well as their contributions to pathological states, such as degenerative disease. In this context, DARC imaging remains a valuable tool, particularly given the growing interests in therapeutics targeting microglia for neuroprotection (Wang and Cepko, 2022; Xiao et al., 2024).

Although pharmacological targeting of microglia in preclinical studies has shown promising results, evidence of clinical efficacy remains limited. For example, Keenan et al. (2024) investigated minocycline, a tetracycline antibiotic known for its anti-inflammatory properties and ability to modulate microglial activity, for treating geographic atrophy. However, minocycline did not significantly alter lesion expansion or prevent vision loss. These findings highlight the need for more representative animal models; for instance, models repopulated with human microglia may provide greater clinical predictability for evaluating therapeutic effects (Ma et al., 2024). Integrating models that utilize human microglia with DARC imaging may offer a more accurate representation of how this subset of microglia behaves in human disease. Furthermore, advancements in adaptive optics could enhance the resolution of *in vivo* retinal imaging, improving the ability to track disease progression and therapeutic response at the cellular level.

Taken together, these findings underscore the need for more precise therapeutic strategies that leverage DARC imaging to identify and target key myeloid cell populations. Such approaches may ultimately pave the way for more effective interventions for neurodegenerative and inflammatory diseases of the eye, with broader implications that could extend to other neurodegenerative conditions of the brain.

*This work was supported [in part] by the Intramural Research Program of the National Institutes of Health (NIH) (to KJM), and also supported by the Office by the Office of the Assistant Secretary of Defense for Health Affairs and the Defense Health Agency J9, Research and Development Directorate, through the Vision Research Program under Award No. (CDMRPL-18-0-VR180205 to KJM and FMN-N). The contributions of the NIH author(s) were made as part of their official duties as NIH federal employees, are in compliance with agency policy requirements, and are considered Works of the United States Government. However, the findings and conclusions presented in this paper are those of the author(s) and do not necessarily reflect the views of the NIH, the U.S. Department of Health and Human Services, or the U.S. Department of Defense.*
